# P-1355. Trends in Carbapenemase and Co-carbapenemase Production among Enterobacterales in Latin America: SMART 2018-2022

**DOI:** 10.1093/ofid/ofae631.1532

**Published:** 2025-01-29

**Authors:** Mark G Wise, Thales Polis, Fakhar Siddiqui, Katherine Young, Mary Motyl, Daniel F Sahm

**Affiliations:** IHMA, Schaumburg, Illinois; MSD Brazil, Sao Paulo, Brazil, Sao Paulo, Sao Paulo, Brazil; Merck & Co., Inc., Rahway, New Jersey; Merck, Rahway, New Jersey; Merck, Rahway, New Jersey; IHMA, Schaumburg, Illinois

## Abstract

**Background:**

The proliferation of carbapenemases in many geographies has compromised the effectiveness of the carbapenem class of antimicrobials and constitutes a major clinical problem. However, carbapenems paired with β-lactamase inhibitors, like imipenem/relebactam (IMR), can restore their activity against isolates carrying some types of carbapenemases. We evaluated trends in carbapenemase and co-carbapenemase production among Enterobacterales collected as part of the SMART surveillance program in Latin America.

**Figure:**
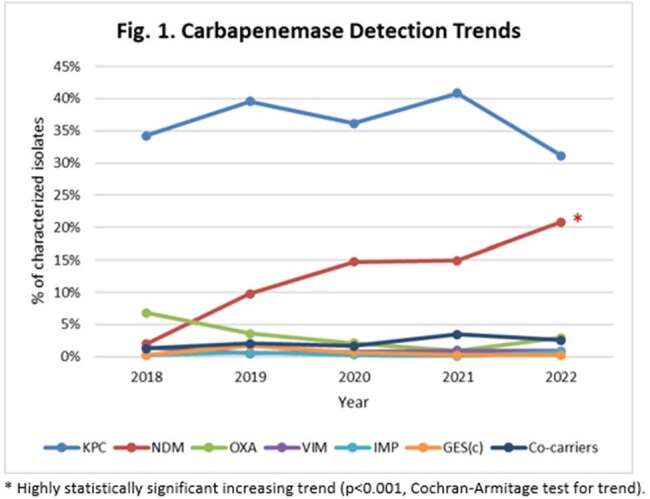

**Methods:**

In 2018-2022, 47 clinical labs in 11 countries in Latin America (Argentina, Brazil, Chile, Colombia, Ecuador, Guatemala, Mexico, Panama, Peru, Puerto Rico, Venezuela) each collected up to 250 consecutive Gram-negative pathogens per year from patients with bloodstream, intraabdominal, respiratory tract, and urinary tract infections. MICs were determined using CLSI broth microdilution and interpreted with 2024 CLSI breakpoints. Most imipenem (IPM) nonsusceptible non-*Morganellaceae* Enterobacterales and ceftolozane/tazobactam (C/T) nonsusceptible Enterobacterales were screened for β-lactamases genes.

**Figure:**
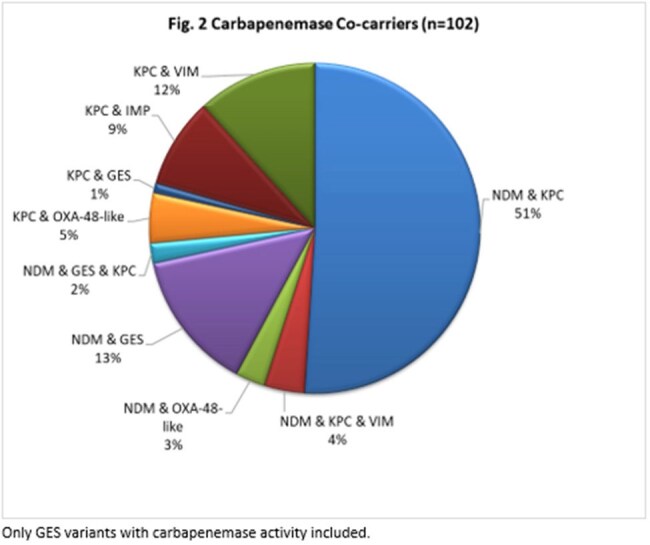

**Results:**

Of the 29,419 Enterobacterales collected, 6669 tested non-susceptible to IPM or C/T; of these, 4474 (67.1%) were characterized molecularly. KPC was the most frequently observed carbapenemase each year, ranging from 40.8% of the characterized isolates in 2021 to 31.1% in 2022, exhibiting an overall slight decreasing trend (Fig. 1). In contrast, the rate of NDM detection increased annually, ranging from 2.0% of the characterized isolates in 2018 to 20.8% in 2022. Rates of detection of other carbapenemases, including OXA-48-like, VIM, IMP and GES remained low and relatively stable over the studied time frame. Detection of carbapenemase co-carriers peaked in 2021 (3.5%), with isolates harboring NDM & KPC the most common genotype (Fig. 2).

**Conclusion:**

KPC was the most commonly encountered carbapenemase among Enterobacterales in Latin America from 2018-2022, suggesting agents that retain potency against KPC-carrying Enterobacterales, like IMR, remain appropriate therapies. However, the increasing trend of NDM detection in this region is of concern as it severely limits treatment options.

**Disclosures:**

**Daniel F. Sahm, PhD**, Pfizer, Inc.: Advisor/Consultant

